# Novel Gas Sensor Signal Acquisition Method: Amplifying Sensor Signals and Enabling Efficient Gas Identification

**DOI:** 10.1002/advs.202415104

**Published:** 2025-04-04

**Authors:** Kangwook Choi, Ryun‐Han Koo, Jinwoo Park, Donghee Kim, Jaehyeon Kim, Hunhee Shin, Gyuweon Jung, Jong‐Ho Lee

**Affiliations:** ^1^ Department of Electrical and Computer Engineering and Inter‐university Semiconductor Research Center Seoul National University Seoul 08826 Republic of Korea; ^2^ School of Transdisciplinary Innovations Seoul National University Seoul 08826 Republic of Korea

**Keywords:** gas diffusion, gas identification, operational method, resistive gas sensor, TCAD simulation

## Abstract

Enhancing sensor sensitivity and gas identification capabilities is essential for the broad application of gas sensors. Developing efficient transducing methods for sensors can be applied to a wide range of sensors. However, developing such methods for resistive sensors remains challenging. In this study, an operating method that enhances both sensitivity and gas identification capability in resistive gas sensors is presented. The sensor operation is divided into two phases: the reaction phase and the signal detection phase, and propose optimized operating methods for each. In the reaction phase, the chemisorption of oxidizing and reducing gases are maximized through appropriate operating methods for each. In the signal detection phase, a read‐bias technique is introduced, enhancing sensitivity across all gases, with a 23‐fold increase for 500 ppb NO_2_ and a sixfold increase for 50 ppm H_2_S. Additionally, the limit of detection (LOD) can be improved, with the NO_2_ LOD reduced from 11.8 to 1.4 ppb. Furthermore, a method for obtaining gas‐specific signal patterns is presented that reflect the unique diffusion properties of each gas by simply adjusting the signal readout conditions. This approach demonstrates the accurate identification of four different gases using only a single sensor.

## Graphical abstract

This study presents a novel method to improve sensitivity and gas identification in resistive gas sensors by dividing operation into reaction and signal detection phases. The reaction phase optimizes chemisorption, while the signal detection phase, using a read‐bias technique, enhances sensitivity. Adjustable readout conditions enable distinct gas identification, allowing precise detection of multiple gases with a single sensor.

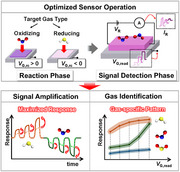

## 1 Introduction

Gas sensors are widely used in various fields, such as air quality monitoring, medical diagnostics, and the detection of toxic gases. Particularly, semiconductor‐type gas sensors have gained significant research interest due to their low cost, possibility of mass production, and compact size.^[^ ^1^, ^2^, ^3^, ^4^, ^5^, ^6^ ^]^ These sensors detect changes in sensing material (such as resistance,^[^ ^7^ ^]^ capacitance,^[^ ^8^ ^]^ or effective charge^[^ ^9^ ^]^) caused by gas reactions and convert them into appropriate electrical signals. Traditionally, research on gas sensors has focused on modifying the properties of the sensing materials to enhance their interaction with gases. For example, traditional methods have employed sensing materials incorporating nanostructures^[^ ^10^, ^11^, ^12^, ^13^ ^]^ or noble metal catalysts^[^ ^14^, ^15^ ^]^ to improve gas response. While these methods offer excellent sensitivity and selectivity, they often face challenges related to CMOS incompatibility and reliability issues.

Improvements in sensor transducing operation, such as optimizing operating bias conditions, can enhance sensor performance even without any engineering to the sensing material.^[^ ^16^, ^17^, ^18^, ^19^, ^20^ ^]^ This approach is highly versatile, as it can be applied to a wide range of sensors and impacts the overall sensor field. However, only a limited number of studies have focused on this area, and most have been confined to sensors using specific types of sensors. Choi et al.^[^ ^21^ ^]^ investigated the gate‐tunable effect in a gas sensor based on a MoS_2_/hBN heterostructure field‐effect transistor. Their study demonstrated that the optimal gate bias for linear sensing signals depends on the electron accepting or donating properties of the target gas, with results applicable to gases such as NO_2_ and NH_3_. Similarly, Meile et al.^[^ ^22^ ^]^ investigated the promotive effect of a pulse biasing scheme on the recovery speed of a field‐effect transistor‐type gas sensor. Their work focused on H_2_S gas sensing, demonstrating that applying a negative pulse bias to the control gate of the sensor significantly improved the recovery speed. Potyrailo et al.^[^ ^23^ ^]^ demonstrated that by applying an impedance‐based biasing technique to conventional semiconducting metal oxides, they could significantly enhance gas sensor performance. This biasing approach, through dielectric excitation measurements, led to a linear gas response, broad detection range, and improved baseline stability.

**Figure 1:**
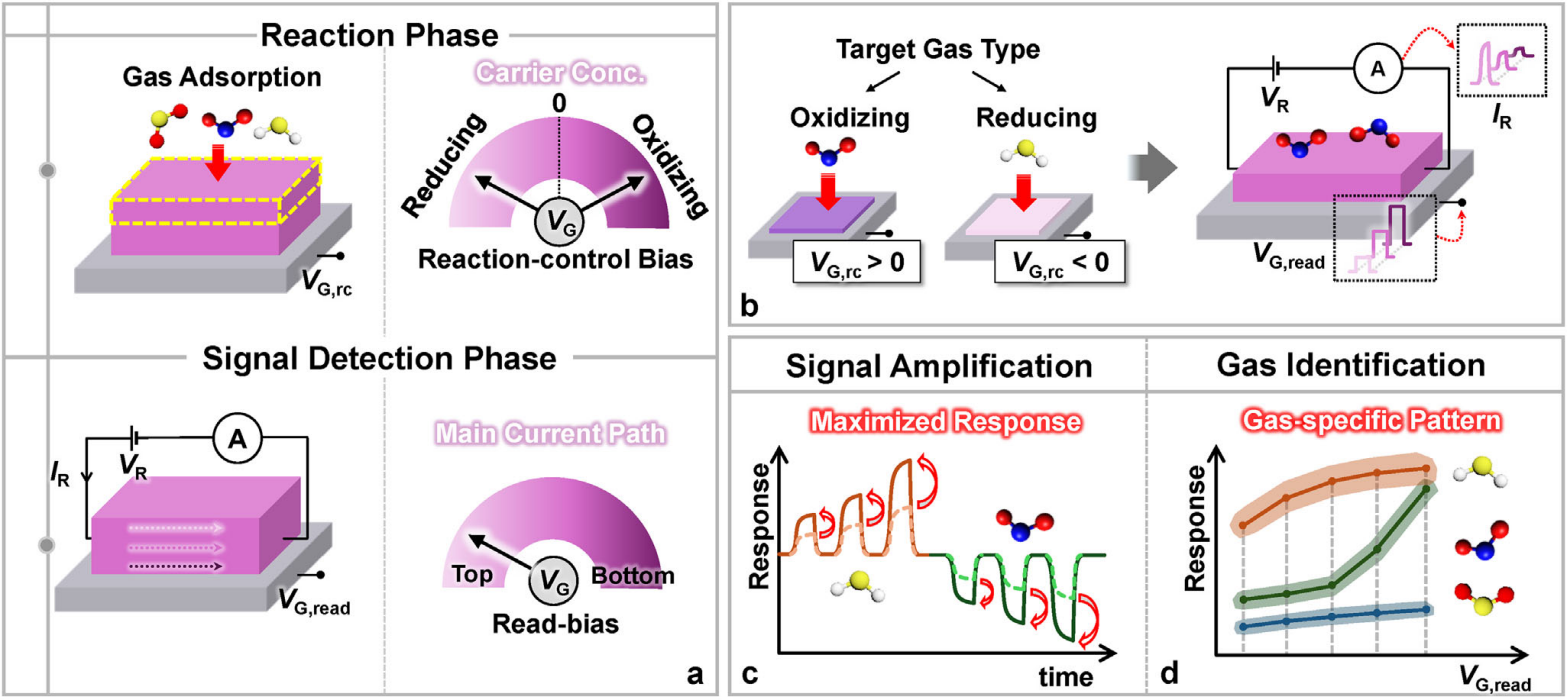
a) Two operation phases for optimizing the performance of resistive gas sensors. b) Reaction‐control bias (*V*_G,rc_) and read‐bias (*V*_G,read_) applied during each operation phase. c) Enhancing sensor response using the read‐bias method. d) Distinguishing gases using the response spectrum based on the read‐bias.

Since most gas sensors are resistive, there is a significant need to develop improved operation methods for these types of sensors. However, there is still a lack of development in the operating methods for these sensors. In this study, we propose an advanced sensor operation method for resistive gas sensors. We systematically divide sensor operation into the reaction phase, where the gas interacts with the sensing material at an elevated temperature, and the signal detection phase, where the electrical signal is obtained at room temperature, and present an optimized biasing method for the two distinct phases (**Figure** **1**a). We propose a customized optimal operation method tailored to whether the target gas is oxidizing or reducing (Figure 1b), demonstrating that this approach effectively amplifies the sensor signal (Figure 1c). Additionally, by operating the sensor in various modes, we can capture diverse signals, creating unique fingerprints for each gas. We demonstrate that these fingerprints enable the successful identification of gases, even when using a single sensor (Figure 1d).

## 2 Results and Discussion

### 2.1 Characterization of the Resistive Gas Sensor with Embedded Micro‐Heater

**Figure 2:**
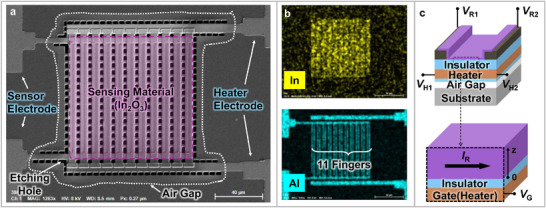
a) Top scanning electron microscope (SEM) image of the sensor used in the experiment. b) Element mapping of the sensor using energy dispersive X‐ray spectroscopy (EDS). c) Electrode structure and cross‐sectional view of the sensor.

**Figure** **2**a shows the scanning electron microscope (SEM) image of the resistive gas sensor with the embedded micro‐heater. The sensor consists of sensor electrodes, sensing material, a *n* ^+^ poly‐Si micro‐heater, and heater electrodes. The sensor electrodes are interdigitated each other and have 11 fingers, each with a length of 90 µm, and the distance between the electrodes is 2 µm. As shown in the energy‐dispersive X‐ray spectroscopy (EDS) mapping image of the sensor (Figure 2b), the sensor electrodes and heater electrodes remain electrically isolated. Figure 2c shows the schematic of the sensor. An air gap with low thermal conductivity is formed below the micro‐heater, preventing heat loss from the heater and enabling efficient energy transfer to the sensing material. Owing to the sophisticated sensor design, the micro‐heater can act as a gate that regulates the carrier concentration in the sensing material. In the next chapter, the gate‐controllability of the micro‐heater will be presented using technology computer‐aided design (TCAD) simulation. The basic gas sensing characteristics of the sensor used in this study will be analyzed in detail in the Methods section to aid in understanding the operation of the sensor.

### 2.2 TCAD Simulation Results

**Figure 3:**
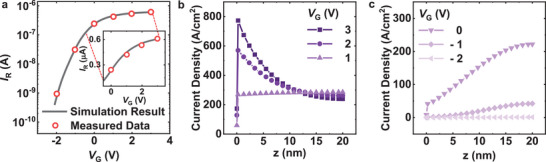
a) Log‐scale *I*_R_‐*V*_G_ data of the sensor (red dotted symbols) and the simulated *I*_R_–*V*_G_ curve (gray line) using TCAD. The inset graph is shown in linear scale. b) c) Vertical profiles of current density across the film cross‐section at various heater biases (*V*_G_ = −2 to 3 V) from TCAD simulations.

By adjusting the voltage (*V*_G_) applied to the micro‐heater, the carrier concentration in the sensing material can be controlled, thereby regulating the sensor current (*I*_R_). Using the cross‐section of the sensor (shown in Figure 2c) as a reference, 2D TCAD simulations were conducted. **Figure** **3**a shows the measured (red dotted symbols) and simulated (gray line) *I*_R_–*V*_G_ of the sensor, taken with a 1 V voltage difference applied between the sensor electrodes. The 2D TCAD simulation results, considering physical parameters such as sensor width and the number of electrode fingers, closely match the experimental data obtained from the actual sensor. Since the sensing material, In_2_O_3_, is an *n*‐type material, the current variation influenced by the *V*_G_ resembles the behavior of an *n*‐type transistor. Figure 3b,c shows the vertical profiles of current density across the film cross‐section (along the *z*‐axis in Figure 2c) at different heater biases (*V*_G_s), as obtained from TCAD simulations. During these simulations, a voltage difference of 1 V was set between the sensor electrodes.

The current flowing through the sensing material can be divided into two main regions: the surface (top) region and the bulk (bottom) region. At *V*_G_ = 0 V, interface traps at the boundary between the sensing material and the insulator on the gate repel electrons from the bulk region, thereby reducing the current density in this area. Applying a gate voltage of 1 V generates an electric field that offsets this effect, promoting an even distribution of current density across the bulk and surface regions. This balance leads to a relatively uniform current density profile from the bulk region to the surface. At lower heater voltages, such as 0 V, the influence of interface traps becomes stronger, depleting the electrons in the bulk region. As a result, the current density in the surface region becomes relatively larger compared to the bulk region. As *V*_G_ increases beyond 1 V, the electric field induces electron accumulation in the bulk region of the sensing material. Consequently, the current density in the bulk region becomes higher than that in the surface region.

The distribution of the current density in the sensing material can be divided into three conditions based on the magnitude of the heater voltage. In the normal condition (*V*_G_ = *V*_N_ = 1 V), the current density is uniformly distributed from the surface region to the bulk region, providing a balanced vertical profile. Under depletion conditions (e.g., *V*_G_ = −1 V), the bulk region undergoes electron depletion, leading to the majority of the current being confined to the surface region. In contrast, under accumulation conditions (e.g., *V*_G_ = 3 V), electrons accumulate in the bulk region, resulting in the majority of the current flowing through the bulk region.

### 2.3 Operating Method for the Resistive Gas Sensor

The primary functions of a gas sensor can be divided into two key roles: the receptor function, where the sensor interacts with gases, and the transducer function, where gas reactions are converted into electrical signals.^[^ ^24^ ^]^ Based on these core functions, we divide the sensor operation into two distinct phases: the reaction phase and the signal detection phase, and we optimize each process accordingly (Figure 1a).

**Figure 4:**
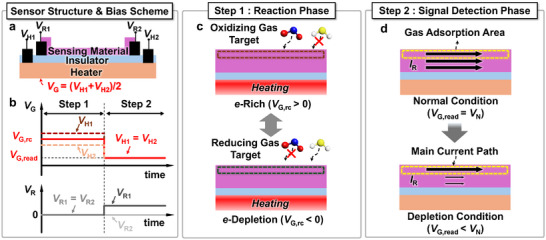
a) Electrode structure of the sensor and b) proposed optimal bias scheme. c) Reaction‐control bias method and heating applied during the reaction phase. Reaction‐control bias method modulates the promotion or suppression of gas reactions during the reaction phase by adjusting the reaction‐control bias and heating conditions. d) Read‐bias method applied during the signal detection phase.

The sensor consists of four electrodes: two sensor electrodes and two heater electrodes (**Figure** **4**a). By adjusting the voltage applied to each of these four electrodes, an operational scheme tailored to each phase can be achieved (Figure 4b). In the reaction phase, the receptor function of the sensor is optimized. The interaction between the sensing material and the gas is determined by the temperature and electron concentration within the sensing material. The operating temperature of the sensing material is determined by the current flowing between the two heater electrodes. This current generates heat, which sets the temperature of the sensing material. Simultaneously, the average of the voltages across heater electrodes acts as a gate voltage (*V*_G_ = (*V*_H1_+*V*_H2_)/2), influencing the electron concentration in the sensing material. A positive gate voltage promotes electron accumulation in the sensing material, thereby increasing reaction to oxidizing gases. Conversely, a negative gate voltage depletes electrons within the sensing material, enhancing the reaction to reducing gases. Considering its role in controlling gas reactions, we have designated the applied bias at this stage as the “reaction‐control bias (*V*_G,rc_)” (Figure 4c).

In the signal detection phase, the focus shifts to the transducer function of the sensor, aiming to convert the prior gas interaction into a suitable electrical signal. The gas reaction in the sensor is reflected as a resistance change in the sensing material. Therefore, applying a fixed voltage difference across the two sensor electrodes (*V*_R1_–*V*_R2_ = 1 V) results in a corresponding change in the current flowing through the sensing material. As demonstrated in the previous TCAD simulation, the gate voltage can adjust the main current path within the sensing material. We categorized the gate voltage conditions into three types—depletion, normal, and accumulation—based on the location of the main current path. By directing this main current path toward the gas‐adsorbed surface region, the sensor response can be amplified. We refer to the bias applied during the signal detection phase as the “read‐bias (*V*_G,read_).” The specific effects of the reaction‐control bias and the read‐bias on sensor performance will be analyzed in detail in the following chapter.

#### 2.3.1 Operating Method in the Reaction Phase

In the reaction phase, the temperature of the sensing material is adjusted, and at the same time, reaction‐control bias is applied to promote the gas reaction. The temperature is controlled by the voltage difference between the two heater electrodes, while the reaction‐control bias is set as the average voltage between them. This reaction‐control bias modulates the electron concentration within the sensing material, thereby promoting or suppressing its interaction with gases.

**Figure 5:**
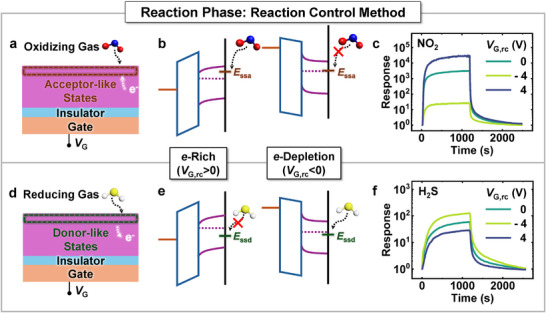
a) Acceptor‐like and d) donor‐like states generated by adsorbed NO_2_ and H_2_S gases, respectively. b) e) Energy band diagrams of the sensor and chemisorption under two different reaction‐control bias conditions. c) f) Response of NO_2_ and H_2_S gases under three different reaction‐control bias voltages.

The mechanism of this bias is based on its ability to control the likelihood of chemisorption through electron concentration modulation.^[^ ^25^ ^]^ For oxidizing gases like NO_2_, when these gases adsorb onto the sensing material, they tend to extract electrons from it, acting as electron acceptors and forming acceptor‐like states (**Figure** **5**a). As a result, the probability of electron trapping in these states increases with higher electron concentrations within the sensing material. Thus, the chemisorption of oxidizing gases is enhanced as electron concentration increases. Applying a positive reaction‐control bias results in electron accumulation, which promotes reactivity with oxidizing gases (Figure 5b). This effect is experimentally confirmed, as the response of 500 ppb NO_2_ increases by approximately 9.17 times at *V*_G,rc_ = 4 V compared to *V*_G,rc_ = 0 V (Figure 5c). Conversely, applying a negative reaction‐control bias depletes electrons, leading to a significant reduction in NO_2_ response.

For reducing gases like H_2_S, which supply electrons as they adsorb onto the sensing material, they act as donor‐like states (Figure 5d). As a result, the probability of electron supply from gas molecules to these states increases as the electron concentration within the sensing material decreases. The chemisorption of reducing gases is therefore enhanced when the electron concentration in the sensing material is lower. Consequently, applying a negative reaction‐control bias depletes electrons within the material, which promotes the chemisorption of reducing gases (Figure 5e). For 50 ppm H_2_S, the response increases by approximately 2.13 times at *V*_G,rc_ = −4 V compared to *V*_G,rc_ = 0 V (Figure 5f). In contrast, a positive reaction‐control bias accumulates electrons within the sensing material, which reduces the probability of taking electrons from reducing gas molecules and subsequently decreases the H_2_S response. These findings demonstrate the effective role of reaction‐control bias in modulating gas interactions, emphasizing the influence of electron concentration on gas reactivity.

#### 2.3.2 Operating Method in the Signal Detection Phase

In the signal detection phase, changes in the sensor due to gas reactions are converted into an electrical signal. Since In_2_O_3_ is an *n*‐type sensing material, it displays an increase in resistance when exposed to oxidizing gases and a decrease when exposed to reducing gases.^[^ ^26^, ^27^ ^]^ By applying a constant voltage across the two sensor electrodes, the current (*I*_R_) flowing through the sensor changes, indicating a gas response.

A primary advantage of the read‐bias method is its capacity to modulate the main current path within the sensing material, enabling more detailed and comprehensive detection. Through the application of read‐bias, multidimensional and increased electrical signals can be obtained from the same gas reaction, significantly enhancing and diversifying the sensor response. These attributes establish read‐bias as an effective approach to advancing sensor performance.

**Figure 6:**
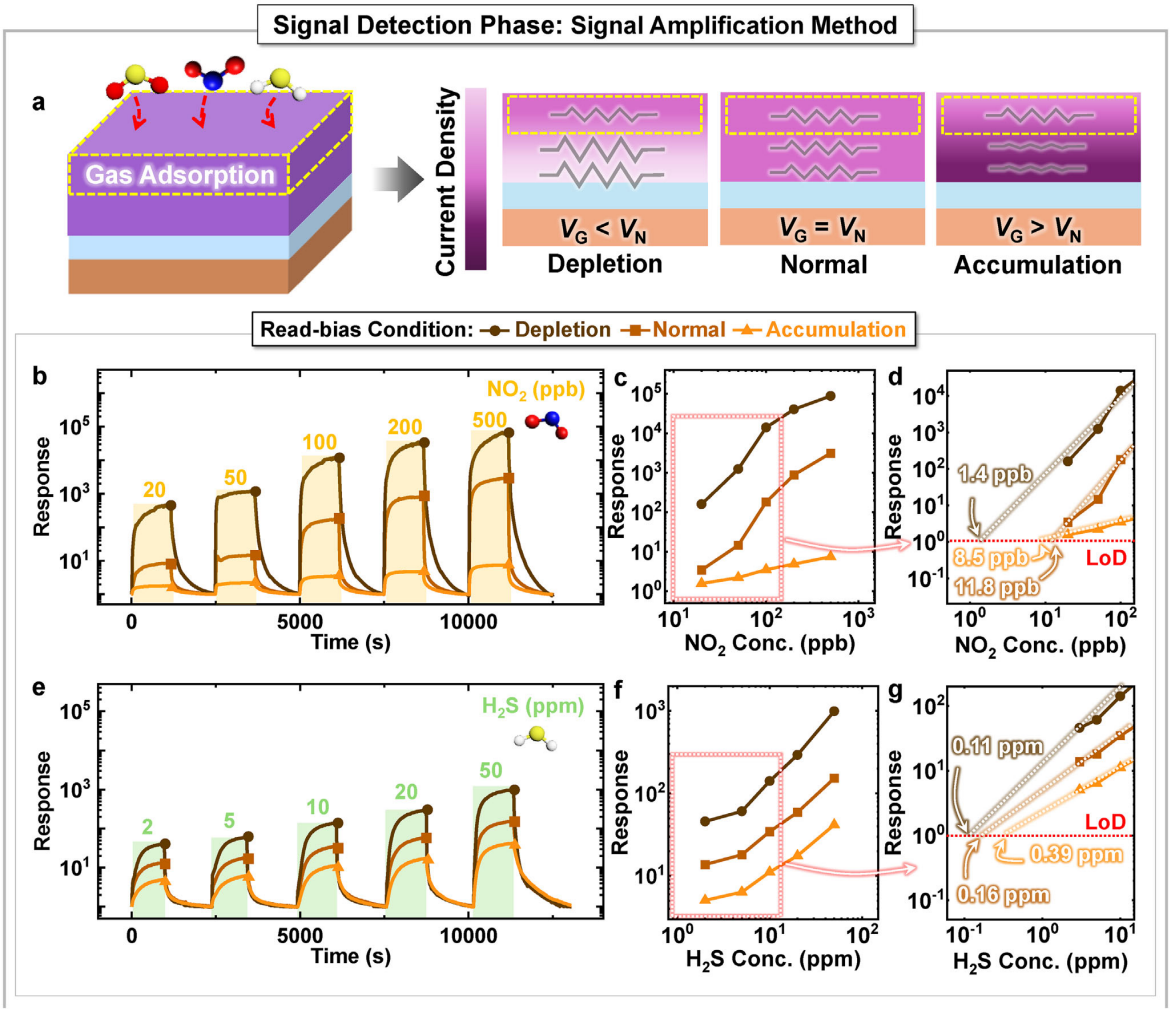
a) Illustration depicting changes in the main current path under varying read‐bias conditions. b) e) Transient responses of the sensor to various concentrations of NO_2_ and H_2_S gases under three different read‐bias conditions. c) f) Response versus NO_2_ and H_2_S gases concentration and d) g) power law fitting line under three distinct read‐bias conditions.

Since most gas molecules adsorb onto the surface of the sensing material, the resistance change due to gas reactions is most evident at the surface (**Figure** **6**a). By directing the dominant current region toward the surface of the sensing material, the sensor response can be maximized. Note that the read‐bias condition allows for adjustment of the dominant current path within the sensing material.

Under normal read‐bias conditions, the current density remains uniform from the surface to the bulk region. On the other hand, when the read‐bias is set to accumulation, the main current path shifts to the bulk region, resulting in most current flowing away from the surface where gas interactions occur, which reduces sensor response. Conversely, with the depletion read‐bias condition, the main current path shifts toward the surface region. This shift directs a larger portion of the current through the area where gas reactions occur, increasing the sensor response. This modulation of the current path by adjusting the read‐bias condition enables diverse signal extraction from the same gas reaction.

Figure 6b,e shows the transient response of the resistive sensor to various concentrations of NO_2_ and H_2_S gases, respectively, under different read‐bias conditions. Under normal conditions (*V*_G,read_ = 1 V), the current flows uniformly from the bulk region to the surface region, meaning that only the current component flowing through the surface region contributes to the gas response. In comparison, under depletion conditions (*V*_G,read_ = 0 V), the current component in the surface region becomes larger relative to that in the bulk region. As a result, most of the current is influenced by the gas, leading to an increased response compared to normal conditions. For instance, for 500 ppb NO_2_ and 50 ppm H_2_S, the responses increase by approximately 23 times and 6 times, respectively, under depletion conditions compared to normal conditions, highlighting the increase effect enabled by this bias adjustment. Conversely, under accumulation conditions, most of the current flows through the bulk region. Since most of the adsorbed gas molecules are located near the surface, the response to the gases decreases compared to normal conditions. The cyclic transient response demonstrates the consistent performance of the read‐bias method over multiple cycles (Figure S1, Supporting Information). This ensures that the proposed method is reliable for enhancing sensor performance in practical applications. Even in the presence of humidity, the sensor maintains a higher response in depletion conditions compared to normal or accumulation conditions (Figure S2, Supporting Information). This trend demonstrates that the depletion condition remains effective in enhancing the gas response despite the presence of humidity.

We investigated the influence of sensing material thickness on the proposed read‐bias effect. As the thickness of the sensing material increases, the influence of gate on the channel diminishes, resulting in a reduced modulation of the current by the read‐bias voltage. Consequently, the impact of the read‐bias condition becomes less pronounced with thicker sensing materials (Figure S3, Supporting Information). To enhance the read‐bias effect, reducing the thickness of the sensing material is advantageous. However, excessively thin sensing materials often lead to increased low‐frequency noise (1/*f* noise), which degrades the signal‐to‐noise ratio (SNR) and compromises sensor reliability.^[^ ^28^ ^]^ Therefore, optimizing the sensing material thickness is crucial to achieving a balance between the read‐bias effect and stable sensor performance. In this study, a 20 nm‐thick sensing material was selected to maintain this balance.

Figure 6d,f shows the response to various concentrations of NO_2_ and H_2_S gases under three different read‐bias conditions, with dashed lines representing linear approximations of the sensor response. The response versus gas concentration data exhibits a strong linear fit on a log–log plot, following a power law relationship.^[^ ^29^ ^]^ The limit of detection (LOD) represents the lowest concentration of gas that the sensor can detect.^[^ ^30^ ^]^ In this study, the LOD for each read‐bias condition is determined by the point where the linear approximation of the sensor response for each gas reaches 1. The LODs for NO_2_ and H_2_S gases are lower under depletion conditions (1.4 ppb, 0.11 ppm) compared to normal conditions (11.8 ppb, 0.16 ppm; Figure 6c,g).

The sensitivity to NO_2_ gas under three different read‐bias conditions (depletion, normal, and accumulation) is 188.89, 6.79, and 0.0118 ppb^−1^, respectively, while the sensitivity to H_2_S gas under those conditions is 20.428, 2.971, and 0.778 ppm^−1^, respectively. For both gases, the sensor exhibits significantly higher sensitivity under depletion conditions compared to other read‐bias conditions. These results highlight the advantage of depletion read‐bias condition for enhancing sensor sensitivity.

#### 2.3.3 Optimization of Operation in Both Phases

The reaction‐control bias and read‐bias each enhance one of the two primary operational functions of the sensor: the receptor function and the transducer function. By organizing the bias scheme into two distinct phases, both bias methods can be effectively utilized, enabling optimal sensor performance. This approach allows for an operational scheme that can be precisely tailored to each target gas, maximizing the response of the sensor.

**Figure 7:**
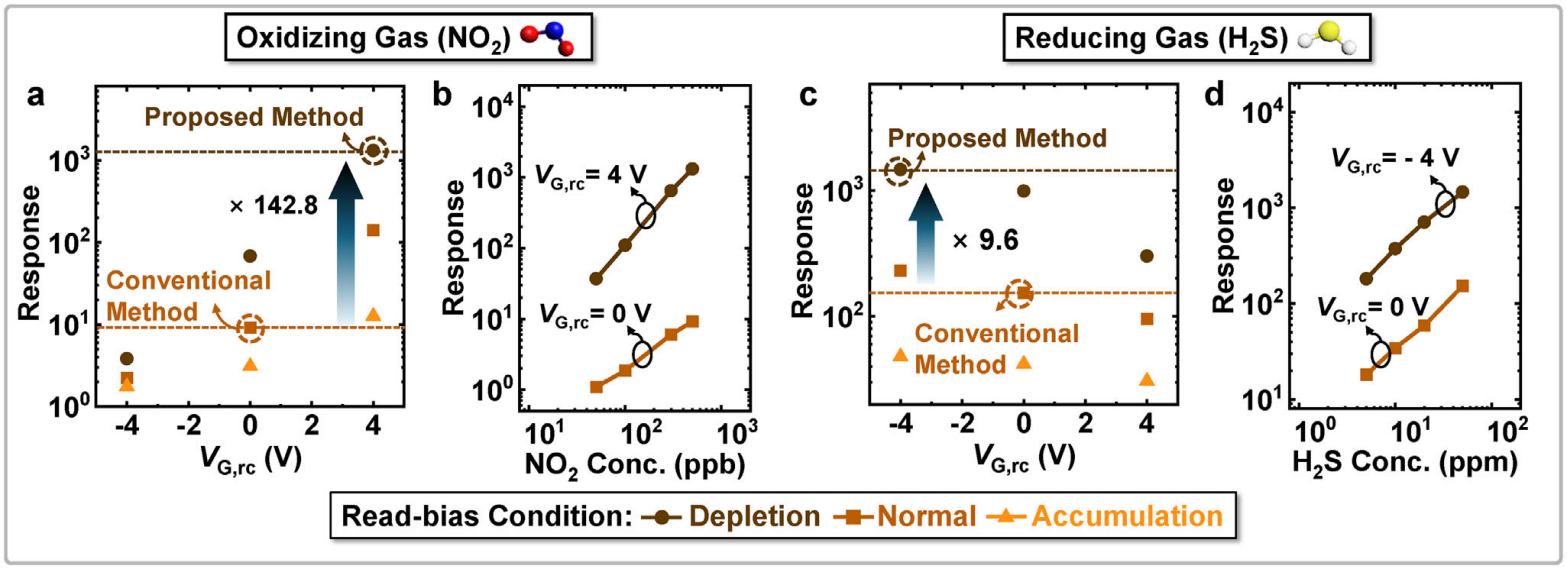
a) c) NO_2_ and H_2_S gas response of the sensor as a function of reaction‐control bias condition (*x*‐axis) as a parameter of read‐bias condition (symbol). b) d) Response versus NO_2_ and H_2_S gas concentration for the proposed method and the conventional method.

**Figure** **7**a,c shows the response to NO_2_ and H_2_S gases, respectively, when the optimized bias scheme using both the reaction‐control bias and read‐bias methods is applied. Since NO_2_ is an oxidizing gas, it shows a higher response under a positive reaction‐control bias (right side of the *x*‐axis in Figure 7a). On the other hand, H_2_S, being a reducing gas, exhibits a higher response under a negative reaction‐control bias (left side of the *x*‐axis in Figure 7c). Both gases also demonstrate higher responses under depletion conditions (circle symbol in Figure 7a,c) of the read‐bias. For NO_2_, the optimized scheme (*V*_G,rc_ = 4 V; Read‐bias Condition: Depletion) results in a 142.8‐fold increase in response compared to the conventional method (*V*_G,rc_ = 0 V; Read‐bias Condition: Normal). Similarly, for H_2_S, the optimized scheme (*V*_G,rc_ = −4 V; Read‐bias Condition: Depletion) results in a 9.6‐fold increase in response compared to the conventional method. The effect of the optimized scheme is observed as an increased response across various concentrations of both gases. Figure 7b,d show the response of NO_2_ and H_2_S at various concentrations using both the proposed optimized scheme (*V*_G,rc_ = 4 V for NO_2_, *V*_G,rc_ = −4 V for H_2_S; Read‐bias Condition: Depletion) and the conventional method (*V*_G,rc_ = 0 V; Read‐bias Condition: Normal). For both gases, the proposed optimized scheme consistently demonstrates a higher response at all concentrations compared to the conventional method.

### 2.4 Gas Identification Using the Response Spectrum

**Figure 8:**
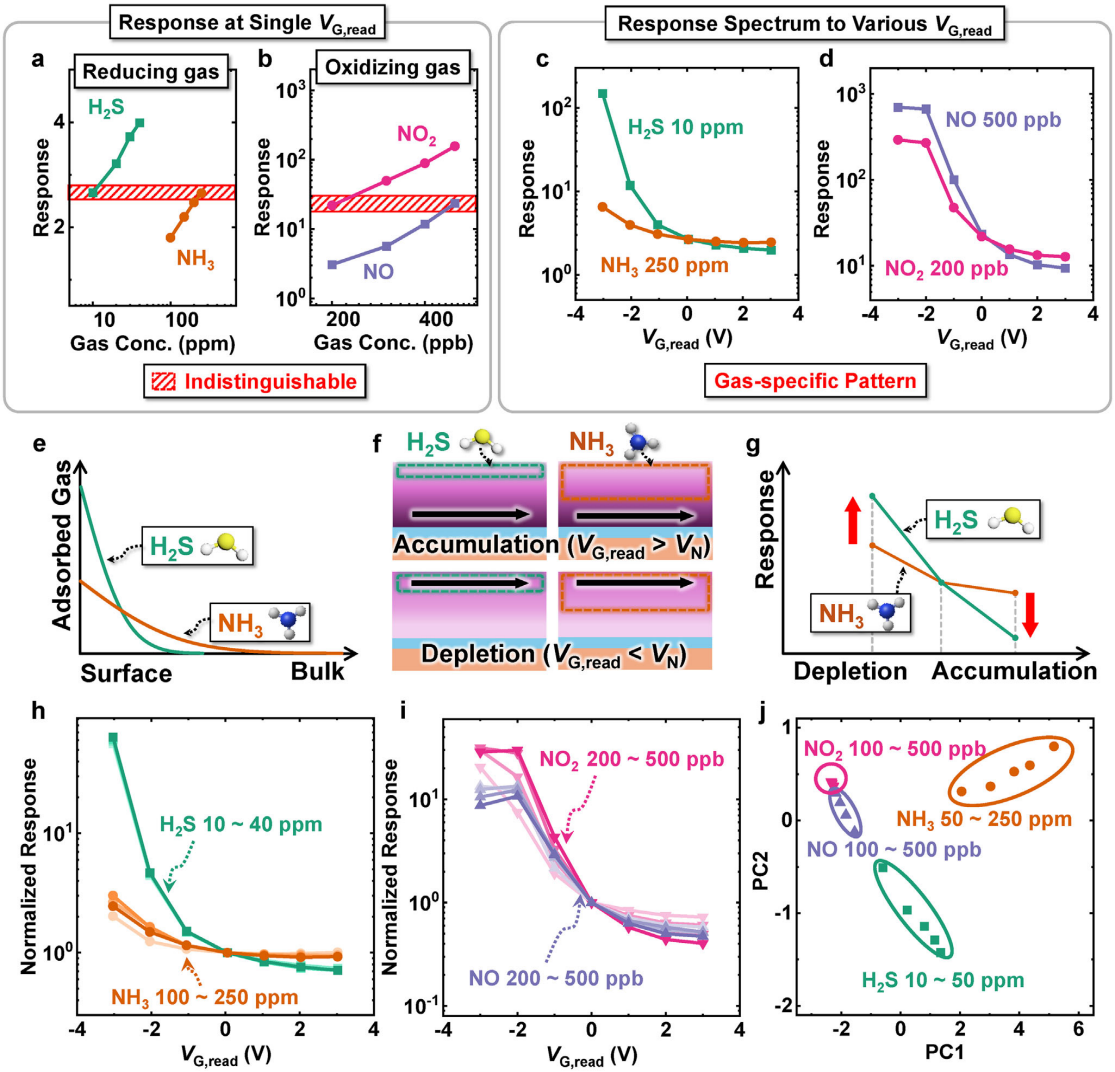
Response versus gas concentration for a) reducing gases (H_2_S, NH_3_) and b) oxidizing gases (NO_2_, NO) at single read‐bias condition. Response spectrum of c) reducing gases (10 ppm H_2_S, 250 ppm NH_3_) and d) oxidizing gases (200 ppb NO_2_, 500 ppb NO) under various read‐bias conditions. e) Diagram of the vertical profile of adsorbed H_2_S and NH_3_ gas concentrations within the sensing material. f) Schematic diagram and g) graph showing the difference in the effect of read‐bias on the response of two gases with different vertical profiles. Normalized response of various concentrations of h) reducing gases (H_2_S, NH_3_) and i) oxidizing gases (NO_2_, NO) under different read‐bias conditions. j) Principal component analysis (PCA) results based on the response spectra of four different gases (H_2_S, NH_3_, NO_2_, NO) under various read‐bias conditions.

**Figure** **8**a,b shows the response of various concentrations of reducing gases (H_2_S, NH_3_) and oxidizing gases (NO_2_, NO) under a single read‐bias condition (*V*_G,read_ = 0 V). Generally, exposure to different gas conditions can exhibit comparable responses, making it difficult to identify them with just one sensor.^[^ ^1^ ^]^ For instance, the response to 10 ppm of H_2_S and 250 ppm of NH_3_ (dashed area in Figure 8a) or 200 ppb of NO_2_ and 500 ppb of NO (dashed area in Figure 8b) is almost identical, which complicates the distinction between these gases using a single device. However, the read‐bias method enables the extraction of additional information from the same gas reaction. This multidimensional data enhances the ability to distinguish between different gases effectively.

Figure 8c compares the responses of 10 ppm H_2_S and 250 ppm NH_3_ at different read‐bias voltages (*V*_G,read_s). At *V*_G,read_ = 0 V, both gases exhibit the same response. However, at *V*_G,read_ > 0, 250 ppm NH_3_ shows a higher response, while at *V*_G,read_ < 0, 10 ppm H_2_S has a higher response. Figure 8d shows the comparison of 200 ppb NO_2_ and 500 ppb NO under various read‐bias voltages (*V*_G,read_s). NO_2_ and NO also show differences in their response spectrum under various read‐bias conditions.

The variation in the response spectrum of each gas to read‐bias condition can be attributed to the vertical profile of adsorbed gases in the sensing material. Gas molecules introduced into a metal oxide semiconductor (MOS) sensing material diffuse inside due to concentration gradients. Gas molecules with smaller molar weights diffuse more rapidly. Once these diffusing gas molecules undergo a reaction, they become immobilized and adsorb onto the sensing material. Consequently, the vertical profile of the adsorbed gas concentration within the MOS film is governed by the gas diffusion coefficient (*D*_k_) and the reaction constant (*k*). A higher diffusion rate and lower reaction rate (i.e., larger *D*_k_/*k*) result in gas molecules penetrating deeper into the sensing material. In contrast, when the diffusion rate is slower, and the reaction rate is higher (i.e., smaller *D*_k_/*k*), the majority of the gas molecules are adsorbed near the surface.^[^ ^29^, ^31^ ^]^

NH_3_, due to its smaller molar weight, diffuses more easily within the sensing material compared to H_2_S.^[^ ^31^ ^]^ Consequently, while most H_2_S molecules are adsorbed and react near the surface, NH_3_ molecules can penetrate deeper into the sensing material (Figure 8e). Thus, for NH_3_ gas, the reduction in reactivity under accumulation conditions is less pronounced than for H_2_S. Conversely, the enhancement in reactivity under depletion conditions, compared to the normal state, is more significant for H_2_S gas (Figure 8g). By analyzing the normalized response of each gas based on its response at *V*_G,read_ = 0 V, it is possible to evaluate the trends in the response spectrum under different read‐bias conditions (Figure 8h). This trend is consistent across all concentration ranges. Although NO_2_ and NO exhibit almost no difference in their normalized response versus *V*_G,read_ graphs, a noticeable difference appears at *V*_G,read_ = −3 V (Figure 8i).

Figure 8j shows the results of principal component analysis (PCA) based on the normalized response. The dataset used for PCA consists of sensor responses to five different concentrations of four distinct gases (NH_3_, H_2_S, NO, and NO_2_). The response spectra are generated under seven different read‐bias conditions (*V*_G,read_ = −3 to 3 V), enabling multidimentional analysis of the gas‐response behavior. The gases are distinctly clustered and separated, demonstrating the capability of the sensor for effective gas discrimination.

Conventional studies have emploied various strategies to enhance gas discrimination capability, including temperature modulation, sensor arrays, and advanced material engineering.^[^ ^32^ ^]^ For example, Tonezzer et al.^[^ ^33^ ^]^ utilized temperature gradients in a single nickel oxide nanowire sensor to generate unique thermal profiles, enabling gas discrimination based on these distinct patterns. However, this method requires precise temperature modulation and control, adding a layer of complexity to the sensing system. Ren et al.^[^ ^34^ ^]^ developed a Ga‐doped In_2_O_3_ sensor array combined with pattern recognition algorithms for gas discrimination. This system achieves excellent selectivity but requires multiple sensors and computationally intensive algorithms, further complicating the sensing setup. Chu et al.^[^ ^35^ ^]^ introduced a sensor array approach combining neural networks to classify gas mixtures with high accuracy. However, the requirement for multiple sensors and extensive data processing increases system complexity. Similarly, Kim et al.^[^ ^36^ ^]^ demonstrated gas mixture identification using gold‐decorated metal oxide sensor arrays coupled with neural networks. While effective in improving selectivity, this approach depends on material modifications and sophisticated data analysis, limiting its practical integration. A detailed quantitative comparison of these approaches is summarized in **Table** **1**.

**Table 1:** Comparison with other gas discrimination technologies from recent studies based on sensing material, concentration range, limit of detection, response time, discrimination method, number of data points, and operating temperature.

	Tonezzer et al. (2018)^[^ ^33^ ^]^	Ren et al. (2022)^[^ ^34^ ^]^	Chu et al. (2021)^[^ ^35^ ^]^	Kim et al. (2023)^[^ ^36^ ^]^	This work
Sensing Material	NiO Nanowires	Ga‐In_2_O_3_ Nanotube	Metal‐oxide Semiconductor^a)^	Au‐decorated SnO_2_, In_2_O_3_, TiO_2_ Film	In_2_O_3_ Film
Concentration Range	0.1–1 ppm (H_2_S), 50–20000 ppm (Ethanol, H_2_, CO, NH_3_, CO_2_, LPG)	0.5–500 ppm (Trimethylamine)	10–50 ppm (NO_2_, CO)	1–50 ppm (NO_2_, CO, NH_3_)	20–500 ppb (NO_2_), 2–50 ppm (H_2_S)
Limit of Detection	Not Specified	13.83 ppb (Trimethylamine)	13.31 ppb (NO_2_), 40.92 ppb (CO)	Not Specified	1.4 ppb (NO_2_), 0.11 ppm (H_2_S)
Response Time (s)	350 (200 °C), <30 (250–400 °C)	1	110–140	Not Specified	121.18
Discrimination Method	Temperature Gradient Response	Sensor Array	Sensor Array, Feature Extraction	Sensor Array, Transient Response	Read‐bias Method
Number of Datapoints^b)^	5×1	1×4	350×4	350×5	7×1
Operating Temperature	200–400 °C	240 °C	290–320 °C	200 °C	200 °C

^a)^ TGS 2600, TGS 2602, TGS 2610, TGS2620 obtained from Figaro Engineering Inc;

^b)^ (Datapoints extracted per sensor) × (Number of sensors).

In contrast, the read‐bias method proposed in this study enables a single sensor to generate a distinct response spectrum for multiple gases under electrical modulations. Utilizing the intrinsic reaction‐diffusion characteristics of gases within the sensing material eliminates the need for temperature modulation, multiple sensing layers, or complex computational algorithms. The PCA results in Figure 8j show clear clustering and separation of gases, highlighting the effectiveness of this approach. Not only does the proposed method simplify the sensing system, but it also enhances robustness and accuracy, providing a practical and scientifically sound solution for gas identification.

## 3 Conclusion

In this study, we proposed and optimized the operating method for resistive gas sensors embedded with a micro‐heater. Considering the key functions of gas sensors—the receptor function and the transducer function—we divided the sensor operation into two phases: the reaction phase and the signal detection phase. During the reaction phase, we focused on facilitating gas‐material interactions by controlling the temperature of the sensing material and applying a reaction‐control bias for the reaction. In the signal detection phase, we applied a read‐bias to achieve more diversified and increased electrical signals.

The principles and effects of the reaction‐control bias and read‐bias were thoroughly examined in each phase. The reaction‐control bias modulates electron concentration within the sensing material, influencing the likelihood of gas chemisorption. Applying this bias increased the responses of NO_2_ and H_2_S gases by approximately 9.17 times and 2.13 times, respectively. Through TCAD simulations, we verified that the read‐bias effectively adjusts the main current path within the sensing material. Based on the dominant current path, we classified the read‐bias conditions into three types: depletion, normal, and accumulation. The depletion condition directs the dominant current path toward the surface, where gas adsorption primarily occurs.

We further proposed an optimized scheme that combines both the reaction‐control bias and read‐bias methods. Under this optimized scheme, responses to NO_2_ and H_2_S gases were approximately 142.8 and 9.6 times greater than those observed with the conventional scheme, demonstrating substantial improvements in sensor response and sensitivity. Finally, the read‐bias approach facilitates accurate gas identification by utilizing the distinct diffusion properties within the sensing material. This approach enables differentiation between four gases (H_2_S, NH_3_, NO_2_, and NO). These findings demonstrate that the proposed operating method not only enhances gas detection but also offers a practical and effective solution for identifying various gases using a single sensor.

## 4 Experimental Section

### Sensor Fabrication

The sensor was produced through the Si CMOS fabrication process. Initially, a 6‐inch *n*‐type Si wafer with (100) orientation underwent standard cleaning. A 550 nm thick SiO_2_ layer was thermally grown using the wet oxidation technique. After that, a 350 nm thick *n* ^+^ poly‐Si layer was deposited by low‐pressure chemical vapor deposition (LPCVD) and patterned to create the heater. A passivation layer comprising SiO_2_/Si_3_N_4_/SiO_2_ (10 nm/20 nm/10 nm) was then sequentially deposited using LPCVD. The contact holes were defined through photolithography and reactive ion etching (RIE). For the metal electrodes, a photoresist was patterned, and a Ti/TiN/Al/TiN (20 nm/20 nm/60 nm/20 nm) layer was deposited using sputtering, followed by a lift‐off process involving acetone immersion and sonication. The H_2_ alloying process was carried out at 400 °C for 10 min. To form the air gap beneath the heater, etching holes were patterned using inductively coupled plasma (ICP) etching with CF_4_ gas. Subsequently, the Si substrate was anisotropically etched using RIE with SF_6_ gas. Finally, 20 nm‐thick In_2_O_3_ was deposited by sputtering and patterned through a lift‐off process. To stabilize the sensing material, annealing was conducted at 300 °C for 30 min in the air.

### Gas‐Sensing Characteristics

The sensor operation was measured using a semiconductor device parameter analyzer (B1500A, Agilent) and a probe station. The target gases (NO_2_, H_2_S, NH_3_, NO) were mixed with dry air in a mixing chamber and injected into the test chamber at a flow rate of 200 sccm, controlled by a mass flow controller. The temperature of the sensing material was controlled by the embedded micro‐heater. The operating temperature was set to 200 °C for target gases. Before gas injection, dry air was introduced into the chamber to remove any residual gases and ensure a stable baseline for the sensing material.

After the gas was introduced, the response of the sensor was calculated based on the type of gas being detected:

(1) $$\mathrm{Response}\;\left(\mathrm{Oxidizing}\;\mathrm{gas}\right)=R_{S}/R_{S0}=\left(V_{R}/I_{R}\right)/\left(V_{R}/I_{R0}\right)=I_{R0}/I_{R}$$

(2) $$\mathrm{Response}\;\left(\mathrm{Reducing}\;\mathrm{gas}\right)=R_{S0}/R_{S}=\left(V_{R}/I_{R0}\right)/\left(V_{R}/I_{R}\right)=I_{R}/I_{R0}$$

where *I*_R_ and *I*_R0_ are the currents flowing through the sensing material of the sensor with and without gas reaction, respectively. The response was determined by the extent to which the current (*I*_R_) varies in comparison to the baseline current (*I*_R0_) that was measured prior to the gas reaction.

The sensitivity was assessed by plotting the sensor response against various concentrations of each gas. A linear fit was applied to the data, and the slope of the resulting plot was extracted as the sensitivity, providing a quantitative measure of the ability to detect changes in gas concentration.

The response time and recovery time of the sensor are dominantly influenced by operating temperature (Figure S4a–c, Supporting Information). To understand this behavior, kinetic modeling of gas‐sensor interactions was conducted using Langmuir theory. By fitting the transient current curves during the response and recovery phases, the adsorption and desorption kinetics underlying the performance of the sensor were analyzed. The extracted time constants for response and recovery at operating temperatures of 100, 160, and 200 °C were (1459.34 and 93.73 s), (200.71 and 46.48 s), and (121.18 and 7.32 s), respectively. At higher temperatures, the response time decreased due to enhanced adsorption kinetics, while the recovery time significantly shortened as increased thermal energy facilitated faster desorption.^[^ ^37^ ^]^

Additionally, the presence of humidity significantly reduces the gas response of the sensor. Water molecules compete with target gas molecules for adsorption sites, leading to decreased surface coverage of the target gas. Furthermore, hydroxyl groups formed from water vapor adsorption introduce excess charge carriers, which neutralize the charge transfer effects that enhance gas sensing. This weakens the response of the sensor, resulting in a noticeable decrease in sensitivity under humid conditions, as observed in the experimental results (Figure 2S, Supporting Information).^[^ ^38^ ^]^

### TCAD Simulation

A 2D TCAD simulation was performed using Synopsys Sentaurus Device tool to analyze the resistive type gas sensor. The parameters used in the simulation were based on the fabrication process and physical structure of the actual sensor. The thickness (*t*_H_) and doping concentration (*N*_H_) of the micro‐heater (or gate) were set to 350 nm and 10^20^ cm^−3^, respectively. The thickness of the passivation layer (SiO_2_/Si_3_N_4_/SiO_2_) was set to 10 nm/20 nm/10 nm, with the relative permittivity of SiO_2_ and Si_3_N_4_ set to 3.9 and 7.3, respectively. The In_2_O_3_ used as the sensing material was set with a thickness (*t*_s_) of 20 nm, a bandgap of 8.9, a dielectric constant of 10, a mobility of 9 cm^2^/V·s, and a carrier concentration of 2 × 10^18^ cm^−3^,^[^ ^39^ ^]^ while the distance between the sensor electrodes was 2 µm. The acceptor‐like interface trap density between the sensing material (In_2_O_3_) and the insulator (SiO_2_) was set to 9 × 10^12^ cm^−2^.^[^ ^40^ ^]^ Based on the above parameters, Poisson's equation was solved to obtain the electrical characteristics of the sensor. Simulations were conducted to analyze the relationship between the current (*I*_R_) flowing through In_2_O_3_ and the voltage applied to the heater, as well as the changes in current density within the sensing material based on the heater voltage.

## Conflict of Interest

The authors declare no conflict of interest.

## Supporting information



Supporting Information

## Data Availability

The data that support the findings of this study are available from the corresponding author upon reasonable request.
