# Supplementary material for: Novel Gas Sensor Signal Acquisition Method: Amplifying Sensor Signals and Enabling Efficient Gas Identification
Source: Adv Sci (Weinh). 2025 Apr 4;12(21):2415104. doi: 10.1002/advs.202415104 (PMC12140347; doi:10.1002/advs.202415104)
Supplement: Supplementary file 1 — Supporting Information [file ADVS-12-2415104-s001.docx]

**Supporting Information**

**Novel Gas Sensor Signal Acquisition Method: Amplifying Sensor Signals and Enabling Efficient Gas Identification**

Kangwook Choi^1^, Ryun-Han Koo^1^, Jinwoo Park^1^, Donghee Kim^1^, Jaehyeon Kim^1^, Hunhee Shin^1^, Gyuweon Jung^1,2*^, and Jong-Ho Lee^1*^

*^1^**Department of Electrical and Computer Engineering and Inter-university Semiconductor Research Center, Seoul National University, Seoul 08826, Republic of Korea*

*^2^School of Transdisciplinary Innovations, Seoul National University, Seoul 08826, Republic of Korea*

* Corresponding author. Tel.: +82-2-880-1727; Fax: +82-2-882-4658.

E-mail address: jhl@snu.ac.kr (J.-H. Lee), gwjung@snu.ac.kr (G. Jung)

This file contains:

Supporting Information Figure S1-S4, Table S1

Supporting Figure 1. a) Cyclic transient response to 500 ppb NO_2_ gas of the sensor under different read-bias conditions. b) Average response (columns) and standard deviation (error bars) for each read-bias condition.

Supporting Figure 2. Sensor response to 500 ppb NO_2_ gas as a function of relative humidity level under different read-bias conditions.

Supporting Figure 3. Normalized responses to 500 ppb NO_2_ of sensors with five different sensing material thicknesses (*t*_s_) under different read-bias conditions. The response for each sensor is normalized to the respective value under normal conditions.

Supporting Figure 4. a-c, Transient responses of the sensor to 500 ppb NO_2_ at 100 °C (a), 160 °C (b), and 200 °C (c), with the red line representing the fitted curve.

Supporting Table 1. Response spectra of four different gases (NH_3_, H_2_S, NO, NO_2_) used in Principal Component Analysis (PCA).

| **Gas** | **Concentration** | **Read-bias (*V*_G,read_)** | | | | | | | |
| --- | --- | --- | --- | --- | --- | --- | --- | --- | --- |
|  |  | **-3 V** | **-2 V** | **-1 V** | **0 V** | **1 V** | **2 V** | **3 V** |  |
| NH_3_ | 50 ppm | 2.7052 | 1.565129 | 1.313536 | 1.235566 | 1.2182 | 1.202251 | 1.20068 |  |
|  | 100 ppm | 3.646061 | 2.236892 | 1.928575 | 1.797739 | 1.758031 | 1.773898 | 1.81432 |  |
|  | 150 ppm | 5.819143 | 3.627465 | 2.508959 | 2.193638 | 2.135608 | 2.105334 | 2.088041 |  |
|  | 200 ppm | 7.424857 | 4.030907 | 2.852011 | 2.469185 | 2.365593 | 2.300884 | 2.289854 |  |
|  | 250 ppm | 6.491055 | 3.950704 | 3.059684 | 2.647917 | 2.49913 | 2.421976 | 2.450927 |  |
| H_2_S | 10 ppm | 148.5768 | 11.77859 | 3.976786 | 2.654753 | 2.276686 | 2.080468 | 1.971842 |  |
|  | 20 ppm | 189.245 | 14.57519 | 4.795765 | 3.20927 | 2.768573 | 2.52728 | 2.378787 |  |
|  | 30 ppm | 227.4723 | 17.24694 | 5.558546 | 3.726897 | 3.111119 | 2.782154 | 2.651403 |  |
|  | 40 ppm | 254.5293 | 18.65384 | 6.047969 | 3.989417 | 3.372522 | 3.037602 | 2.844165 |  |
|  | 50 ppm | 297.8787 | 21.62875 | 6.742707 | 4.440683 | 3.710007 | 3.340592 | 3.109556 |  |
| NO | 100 ppb | 0.048914 | 0.125711 | 0.368374 | 0.534835 | 0.603667 | 0.642568 | 0.660241 |  |
|  | 200 ppb | 0.015903 | 0.043549 | 0.170033 | 0.325439 | 0.385724 | 0.431626 | 0.447705 |  |
|  | 300 ppb | 0.005939 | 0.010727 | 0.069563 | 0.1771 | 0.236022 | 0.278718 | 0.292187 |  |
|  | 400 ppb | 0.002682 | 0.003024 | 0.02562 | 0.084615 | 0.134856 | 0.156655 | 0.171782 |  |
|  | 500 ppb | 0.001492 | 0.001429 | 0.009912 | 0.042763 | 0.074134 | 0.097162 | 0.106073 |  |
| NO_2_ | 100 ppb | 0.003411 | 0.003703 | 0.020982 | 0.045644 | 0.064171 | 0.074932 | 0.078431 |  |
|  | 200 ppb | 0.002638 | 0.008764 | 0.033588 | 0.041882 | 0.057266 | 0.068798 | 0.070526 |  |
|  | 300 ppb | 0.00162 | 0.0015 | 0.00737 | 0.0201 | 0.0308 | 0.0372 | 0.0394 |  |
|  | 400 ppb | 0.00107 | 0.000917 | 0.00375 | 0.0113 | 0.0168 | 0.0195 | 0.0218 |  |
|  | 500 ppb | 0.000735 | 0.000595 | 0.0022 | 0.00639 | 0.0101 | 0.0128 | 0.0136 |  |
